# Impact of closed-off management due to COVID-19 rebound on maternal depression during pregnancy

**DOI:** 10.1186/s12884-024-06285-6

**Published:** 2024-01-29

**Authors:** Wanqing Xiao, Yuting Yang, Huiyun Xiao, Peiyuan Huang, Dongmei Wei, Yingfang Wu, Jia Yu, Jian-Rong He, Xiu Qiu

**Affiliations:** 1 Division of Birth Cohort Study, Guangzhou Women and Children’s Medical Center, Guangzhou Medical University, No. 9 Jinsui Rd., Tianhe District, Guangzhou, 510623 Guangdong China; 2 Department of Women’s Health, Guangdong Provincial Key Clinical Specialty of Woman and Child Health, Guangzhou Women and Children’s Medical Center, Guangzhou Medical University, Guangzhou, 510623 China; 3 Guangdong Provincial Clinical Research Center for Child Health, Guangzhou Women and Children’s Medical Center, Guangzhou Medical University, Guangzhou, 510623 China; 4 Provincial Key Laboratory of Research in Structure Birth Defect Disease and Department of Pediatric Surgery, Guangzhou Women and Children’s Medical Center, Guangzhou Medical University, Guangzhou, 510623 Guangdong China

**Keywords:** COVID-19, Lockdown, Closed-off measures, Depression, Pregnant women

## Abstract

**Background:**

This study aimed to assess the impacts of closed-off measures with different strictness levels (lockdown, partial lockdown and non-lockdown) and geographic proximity to patients with coronavirus disease 2019 (COVID-19) on prenatal depression during an epidemic rebound of COVID-19.

**Methods:**

This was a cross-sectional web-based survey including 880 pregnant women. Depressive symptoms were measured by Self-Rating Depression Scale (SDS) and geographic proximity was calculated using Geographic Information Systems. Linear and logistic regression were used to assess the associations of closed-off measures and geographic proximity with SDS scores and depressive symptoms. Restricted cubic splines were used to model non-linear associations between geographic proximity and depression symptoms.

**Results:**

Compared with those living in non-lockdown areas, women in lockdown areas had higher SDS scores (adjusted β: 3.51, 95% CI: 1.80, 5.21) and greater risk of depressive symptoms (adjusted OR: 4.00, 95% CI: 2.18, 7.35), but evidence for partial lockdown was not obvious. A progressive increase in the risk of depressive symptoms was found with decreasing distance to COVID-19 patients when geographic proximity was <8 kilometers. Compared to those in the 5^th^ quintile of geographic proximity, women in the first, second and third quintiles had at least 6 times higher risk of depressive symptoms.

**Conclusions:**

Pregnant women under strict closed-off management during COVID-19 epidemic have high risk of depression. A specific range around the residences of reported COVID-19 patients should be underlined as potential clustering of high prenatal depression levels. Our findings highlight the importance of enhancing mental health management during the COVID-19 epidemic for pregnant women.

**Supplementary Information:**

The online version contains supplementary material available at 10.1186/s12884-024-06285-6.

## Background

Since the first case of coronavirus disease 2019 (COVID-19) reported in December 2019 in Wuhan, Hubei Province, China, the pandemic has lasted for a long time worldwide. The total global number of COVID-19 cases has reached over 670 million, with over 6 million deaths until January, 2023 [1]. Apart from the patients, COVID-19 has also brought a great threat to the health of those uninfected, including a substantial increase of major depressive disorder with additional 53.2 million cases in 2020 due to the pandemic [2]. Quarantine is one of the several common public measures to prevent the spread of contagious diseases. Previous studies in influenza, Ebola and coronavirus diseases, including the severe acute respiratory syndrome and the Middle East respiratory syndrome, have shown the psychological consequence of quarantine in general residents or specific populations such as health care workers [3]. Since the outbreak of COVID-19, the severe acute respiratory syndrome coronavirus 2 (SARS-CoV-2) has experienced three pandemic phases with the toxicity and transmissibility keep changing. The prevention and control measures for COVID-19 have been adapted from strict mass quarantine and citywide lockdown to various loosened measures. Some studies indicated the negative impact of lockdown or quarantine measures on psychological health and a decrease in psychological distress following the loosening of lockdown measures in the general population during the COVID-19 pandemic [4–6].

The vulnerable population, including pregnant women, are susceptible to pandemic-related stress, which may lead to adverse psychological impacts [7, 8]. A systematic review with meta-analysis reported that the overall prevalence of prenatal depression was 25% (95% CI: 20%, 31%) during the COVID-19 pandemic [9]. Besides the risk of being infected themselves, pregnant women may also be worried about the health of their fetuses due to the constraints in antenatal care services and social isolation [10, 11]. Some previous studies compared the depression levels in pregnant women before and during the COVID-19 pandemic. A study conducted in 725 American pregnant women found that women during the pandemic had nearly twice the risk of having possible depressive symptoms than matched women prior to the pandemic [7]. Another two studies in the Netherlands reported no increase in prenatal depressive symptoms during the pandemic [12, 13]. Two studies in China examined the psychological impact of lockdown measures in pregnant women [14, 15]. One of them reported an indirect effect of lockdown on mental health problems of pregnant women (β: 0.03, 95% CI: 0.02, 0.03), while another study indicated that lockdown policy was negatively associated with prenatal depressive symptoms (β: -0.93, 95% CI: -1.51, -0.36). Up to now there are insufficient evidence on the differential effect of closed-off measures with different strictness levels in the era of the ongoing pandemic or potential risk of rebound.

Proximity to the residential locations of COVID-19 patients may be another factor with potential influences on individuals’ mental health status. A study in Bangladesh identified high depression levels in areas where the reported number of COVID-19 cases was particularly high [16]. Another study reported increased anxiety levels in people who knew someone diagnosed with COVID-19 or living in the same neighborhood or town [17]. However, no evidence has been shown about the quantitative interpretation of geographic proximity to COVID-19 patients on the mental health in pregnant women, which would have implications for the targeted psychological interventions at the population level.

There were several local transmissions of the SARS-CoV-2 Delta Variant in some cities of China during 2021, including the rebound in May and June 2021, Guangzhou [18, 19]. The Guangzhou government carried out closed-off measures with different strictness levels and rapidly controlled the epidemic, while the psychological condition of pregnant women in this status was unknown. The objectives of this study were to compare the prenatal depression status among pregnant women under closed-off measures with different strictness levels and to examine the associations of geographic proximity to COVID-19 patients with prenatal depression during an epidemic rebound of COVID-19 in Guangzhou, China. We hypothesized that strict closed-off management would increase the risk of depression during pregnancy, and shorter distances from the residence addresses of COVID-19 patients would result in higher depression risk with a linear or non-linear relation. This study would provide a new perspective to understand the roles of strictness levels of closed-off measures and geographic proximity to COVID-19 patients in identifying pregnant women with high depression levels during an epidemic rebound of COVID-19.

## Methods

### Study design and participants

This is a cross-sectional study conducted during June 3-10, 2021 when closed-off measures had been implemented. Data of participants were collected from a web-based platform, “Sui-Hao-Yun” in Guangzhou, China. This platform is a real-time interaction platform between pregnant women, community health service centers and maternity hospitals with multiple application scenarios, which is constructed by the Municipal Health Commission and Guangzhou Women and Children’s Medical Center, and used for the remote management of pregnant women, especially during the rebound of the COVID-19 epidemic in May and June 2021, Guangzhou, China.

All the pregnant women who registered in the web-based platform during the study period, lived in districts with COVID-19 cases (i.e. Liwan, Haizhu, Yuexiu, Panyu and Nansha) and had the information of depressive symptoms during pregnancy were included in the present study. This study was approved by the Guangzhou Women and Children’s Medical Center Ethics Committee.

### Demographic characteristics

Basic characteristics of participants were self-reported by each pregnant woman through the web-based platform, including age, pre-pregnancy weight and height, last menstrual period (LMP), places of household registration, as well as detailed residence addresses. Pre-pregnancy body mass index (BMI) was calculated by weight before pregnancy in kilograms divided by the square of height in meters, and then classified into four categories according to standard based on Chinese adult population [20]: underweight (<18.5 kg/m^2^), normal weight (18.5–23.9 kg/m^2^), overweight or obese (≥24.0 kg/m^2^). Gestational age was determined from the LMP. Places of household registration were classified into the following groups: Guangzhou city, other cities besides Guangzhou in Guangdong province, and other provinces besides Guangdong.

### Closed-off measures and geographic proximity

For the control of local transmission of the SARS-CoV-2 Delta Variant occurred in Liwan District, Guangzhou in 2021, the Guangzhou government rapidly carried out hierarchical and classified closed-off management, including lockdown and partial lockdown measures, while none closed-off measure was conducted in other areas. In this context, we have a chance to assess the influence of closed-off measures with different strictness levels on the mental health of pregnant women. There were 3 streets in Liwan District determined as lockdown areas, while other 6 streets in Liwan District and some small areas in Haizhu, Yuexiu, Panyu and Nansha Districts were determined as partial lockdown areas along with the progress of epidemic situation, based on the magnitude of COVID-19 epidemic in the corresponding communities and surrounding areas. The detailed information on these measures has been presented in Additional file 1**.**

We used Geographic Information Systems (GIS) spatial analysis techniques to evaluate the geographic distance from the residence address of each pregnant women to those of COVID-19 patients. GIS is a management system for geographic data, which has been applied to solve public health issues [21]. In the present study, we geocoded pregnant women’s residence addresses into geographic coordinates using the GCJ-02 coordinate system of China National Bureau of Surveying and Mapping, and then calculated the distance between two geographic coordinates along the surface of the earth by GIS Arc-GIS 10.2.2 software (Environmental Systems Research Institute, Redlands, Calif.). Residence addresses of all the COVID-19 patients were acquired from the government notices, and we only calculated the geographic distance to the cases announced before the information collection of each pregnant woman.

### Prenatal depressive symptoms

Prenatal depressive symptoms were assessed using the Self-Rating Depression Scale (SDS) compiled by Zung, which is a 4-point scale with 20 items and has been validated in the Chinese population [22, 23]. The Chinese version of SDS has been widely used in women during pregnancy [24–29] and showed good internal consistency [30]. Pregnant women filled out the scale on the web-based platform and reported their perceived frequency of each symptom in the past week corresponding to scores of 1 to 4. The total score was obtained by summing the score of each item together and then multiplying by 1.25. The depressive symptoms were determined as the total score reached or exceeded a cut-off of 53, which has been suggested by previous studies [24, 26–28, 30].

## Statistical analyses

Basic characteristics were presented as means and standard deviations for continuous variables, and frequencies and percentages for categorical variables. Differences in the distributions of basic characteristics among women in groups of closed-off management with different strictness levels were analyzed using ANOVA (continuous variables) or Chi-square test (categorical variables).

Linear regression models were used to evaluate the relationship between SDS scores and closed-off measures with different strictness levels. Logistic regression models were used to estimate the odds ratios (ORs) and their confidence intervals (CIs) for associations of prenatal depressive symptoms with closed-off measures with different strictness levels and quintiles of geographic proximity to the COVID-19 patients. Restricted cubic spline regression was constructed to obtain insights into the linearity of associations between geographic proximity to COVID-19 patients and the risk of having depressive symptoms. We adjusted for maternal age, pre-pregnancy BMI, gestational age and place of household registration in all the adjusted models. We additionally adjusted population density in the sensitivity analysis for examining the association between geographic proximity and depression in the logistic regression model, considering the potential influence of different population density at the residential locations of pregnant women on the association we studied. The population density is defined as the number of population per 1*1 km^2^ in each street and then classified into quartiles, using the data from the Seventh National Population Census of China, 2020. All analyses were performed using SAS version 9.4 (SAS Institute, Cary, NC, USA). *P*<0.05 was considered statistically significant.

## Results

This study included a total of 937 pregnant women and we excluded those without information on residence address (*n*=57), leaving 880 pregnant women for the final analysis. The basic characteristics of pregnant women under closed-off measures with different strictness levels are presented in Table 1. The mean age of the pregnant women was 30.3 ± 4.5 years, and the mean pre-pregnancy BMI was 21.2 ± 3.1 kg/m^2^. The distributions of age and pre-pregnancy BMI were similar across the three areas with lockdown, partial lockdown and non-lockdown measures. The proportion of women at the first trimester in the lockdown group is the highest among the three groups; on the other hand, the proportion of women at the third trimester is the lowest in non-lockdown group. Half of the women reported Guangzhou city as their places of household registration, while this proportion in non-lockdown group was the highest among the three groups.

**Table 1:** Descriptive statistics for the sociodemographic variables of pregnant women

Characteristics	Total	Lockdown (*n*=132)	Partial lockdown (*n*=224)	Non-lockdown (*n*=524)	*P* value
	*n* (%)	*n* (%)	*n* (%)	*n* (%)	
**Age (years), mean ± SD**	30.30 ± 4.52	30.25 ± 5.22	30.12 ± 4.19	30.39 ± 4.47	0.751
< 30	459 (52.16)	72 (54.55)	118 (52.68)	269 (51.34)	0.712
30~34	301 (34.20)	39 (29.55)	79 (35.27)	183 (34.92)	
≥ 35	120 (13.64)	21 (15.91)	27 (12.05)	72 (13.74)	
**Pre-pregnancy BMI (kg/m**^**2**^**), mean ± SD**	21.15 ± 3.09	21.54 ± 3.05	20.90 ± 2.80	21.16 ± 3.21	0.168
<18.5	161 (18.30)	27 (20.45)	40 (17.86)	94 (17.94)	0.608
18.5-23.9	585 (66.48)	81 (61.36)	155 (69.20)	349 (66.60)	
≥24.0	134 (15.23)	24 (18.18)	29 (12.95)	81 (15.46)	
**Gestation**					
1^st^ trimester	136 (15.45)	27 (20.45)	37 (16.52)	72 (13.74)	0.046
2^nd^ trimester	408 (46.36)	62 (46.97)	114 (50.89)	232 (44.27)	
3^rd^ trimester	336 (38.18)	43 (32.58)	73 (32.59)	220 (41.98)	
**Place of household registration**					
Guangzhou city	440 (50.00)	55 (41.67)	77 (34.38)	308 (58.78)	<0.001
Other cities in Guangdong province besides Guangzhou	298 (33.86)	46 (34.85)	103 (45.98)	149 (28.44)	
Other provinces besides Guangdong	142 (16.14)	31 (23.48)	44 (19.64)	67 (12.79)	

Abbreviation: *SD* standard deviation

The SDS scores and the percentages of having depressive symptoms in three groups under closed-off measures with different strictness levels during the first, second and third trimester of gestation are shown in Additional file 2. Pregnant women in the lockdown group reported the highest SDS scores (*P*<0.001) and percentage of having depressive symptoms (*P*<0.001) in the whole population. When considering the different trimesters, there were significant differences in both SDS scores and depressive symptoms among the three groups in women at middle and late pregnancy.

Table 2 shows the results of multivariate regression analysis for the effect of closed-off management with different strictness levels on SDS scores and depressive symptoms in pregnant women. Compared with those living in non-lockdown areas, pregnant women in lockdown areas had higher SDS scores in crude model (unadjusted β: 3.92, 95% CI: 2.20, 5.64) and in adjusted model (adjusted β: 3.51, 95% CI: 1.80, 5.21) in the adjusted model. Pregnant women in lockdown areas also had higher odds of having depressive symptoms than those living in non-lockdown areas in crude model (unadjusted OR: 4.26, 95% CI: 2.35, 7.70) and in adjusted model (adjusted OR: 4.00, 95% CI: 2.18, 7.35). No difference was observed in SDS scores between women living in partial lockdown areas and those in non-lockdown areas (unadjusted β: 1.28, 95% CI: -0.13, 2.69; adjusted β: 0.92, 95% CI: -0.49, 2.33). The association between partial lockdown and depressive symptoms in crude model (unadjusted OR: 1.88, 95% CI: 1.03, 3.44) disappeared after adjustment (adjusted OR: 1.82, 95% CI: 0.97, 3.39).

**Table 2:** Regression analysis for the effects of different closed-off measures on prenatal depression

	SDS scores			Depressive symptoms		
	mean ± SD	Unadjusted model	Adjusted model ^a^	*n* (%)	Unadjusted model	Adjusted model ^a^
		β (95% CI)	β (95% CI)		OR (95% CI)	OR (95% CI)
**Closed-off measures**						
Non-lockdown	38.56 ± 8.54	Ref.	Ref.	26 (4.96)	Ref.	Ref.
Partial lockdown	39.84 ± 9.12	1.28 (-0.13, 2.69)	0.92 (-0.49, 2.33)	20 (8.93)	1.88 (1.03, 3.44)	1.82 (0.97, 3.39)
Lockdown	42.48 ± 10.48	3.92 (2.20, 5.64)	3.51 (1.80, 5.21)	24 (18.18)	4.26 (2.35, 7.70)	4.00 (2.18, 7.35)

Abbreviation: *SD* standard deviation. *OR* odds ratio. *CI* confidence interval

^a^ adjusted for age, pre-pregnancy BMI, gestational weeks and place of household registration

**Fig. 1:**
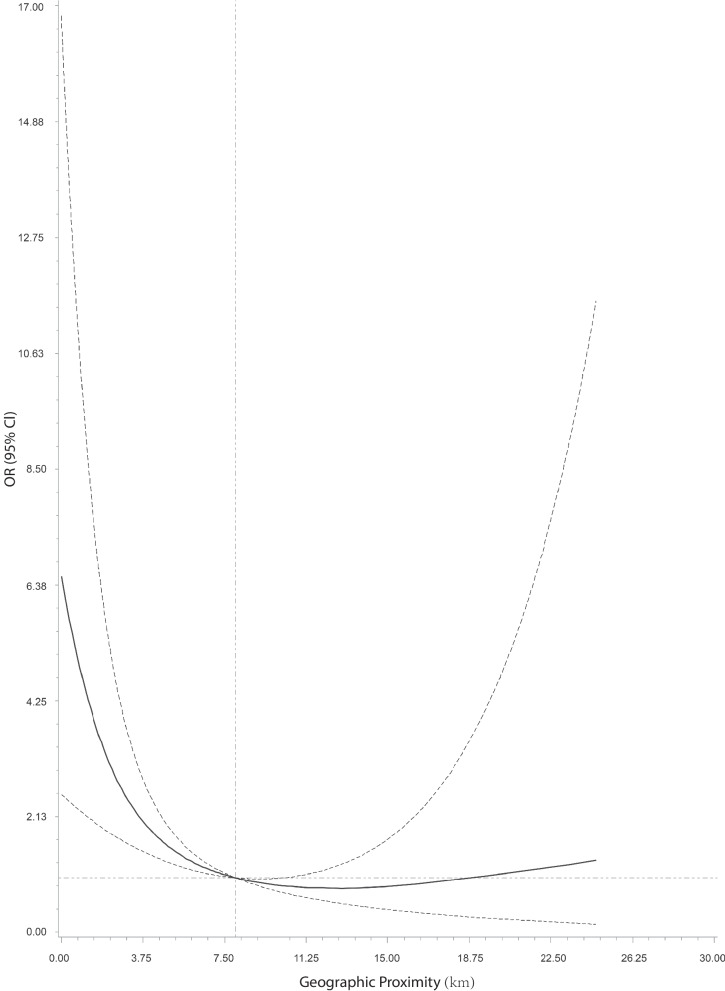
Association between geographic proximity and prenatal depression using restricted cubic spline regression. The associations are presented as risk ratios (ORs, solid line) and the 95% CIs (dashed line) using logistic regression after adjusting for age, pre-pregnancy BMI, gestational weeks and place of household registration. The value of geographic proximity to COVID cases at 8 km was chosen as reference. Knots were placed at the 5^th^, 50^th^, and 95^th^ percentiles of geographic proximity. CI, confidence interval

**Fig. 2:**
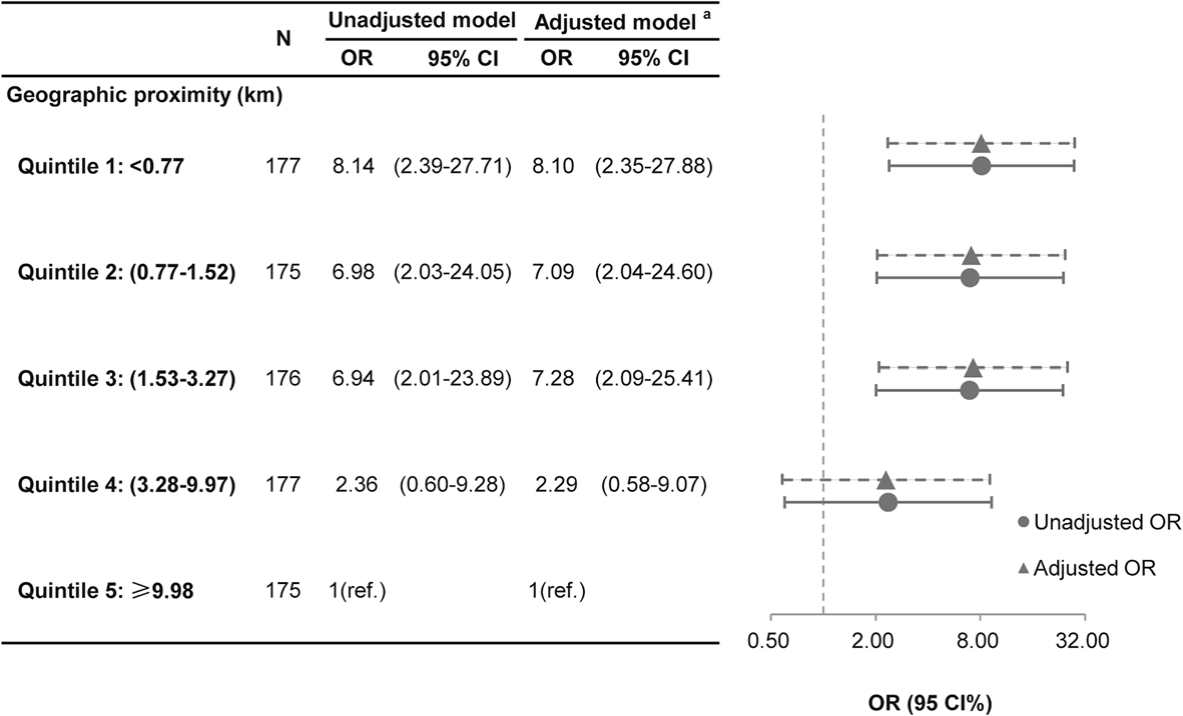
Effect estimates from logistic regression model for association between geographic proximity and prenatal depression. ^a^ adjusted for age, pre-pregnancy BMI, gestational weeks and place of household registration

The associations between geographic proximity and prenatal depressive are shown in Figs. 1 and 2. We found a L-shaped relation between geographic proximity to the residence locations of COVID-19 patients and the risk of having depression symptoms using restricted cubic spline regression. A progressive increased risk of depression during pregnancy was presented with decreasing distance to COVID-19 patients when geographic proximity was less than 8 km. By contrast, the risk of depression remained stable when geographic proximity was more the 8 km (Fig. 1). We further explored the association between depressive symptoms and categories of geographic proximity to COVID-19 patients. Compared with women in quintile 5 (nearest distance to COVID-19 patients: ≥9.98 km), pregnant women in quintile 1 (nearest distance to COVID-19 patients: <0.77 km), quintile 2 (nearest distance to COVID-19 patients: 0.77-1.52 km) and quintile 3 (nearest distance to COVID-19 patients: 1.53-3.27 km) had higher odds of having depressive symptoms with unadjusted ORs (95% CI) of 8.14 (2.39, 27.71), 6.98 (2.03, 24.05) and 6.94 (2.01, 23.89) and adjusted ORs (95% CI) of 8.10 (2.35, 27.88), 7.09 (2.04, 24.60) and 7.28 (2.09, 25.41), respectively (Fig. 2). Pregnant women in quintile 4 had similar odds of having depressive symptoms with those in quintile 5 (Fig. 2). Sensitivity analysis showed similar results when additionally adjusted population density in the logistic regression model (Additional file 3).

## Discussion

In this study of potential mental health problems during the rebound of the COVID-19 epidemic in pregnant women, we found that both the SDS scores and percentage of having depressive symptoms changed with different strictness levels of closed-off management, with a significantly higher SDS scores and risk of having depression symptoms in pregnant women living in lockdown area compared with those in non-lockdown areas. Geographic proximity to COVID-19 patients was negatively associated with an increased risk of having depression symptoms during pregnancy when the nearest distance to COVID-19 patients was less than 8 km. In addition, pregnant women in the first (<0.77 km), second (0.77-1.52 km) and third (1.53-3.27 km) quintiles of geographic proximity had at least 6 times higher risk of having depressive symptoms than those living in the 5^th^ quintile with the nearest distance to COVID-19 patients of ≥9.98 km.

This study presented that closed-off measures with different strictness levels had differential relationships with both binary and continuous indicators of depressive symptoms in pregnant women. Compared with those in non-lockdown areas, women living in lockdown areas had a higher risk of prenatal depression. This finding was supported by a previous study in the general population [4]. Lockdown measures may increase the risk of depression through maladaptive cognitive responses, with the COVID-19 epidemic as a great stressful event [31]. Pregnant women often have more need of routine medical care for the well-beings of themselves and babies, and are more medically vulnerable for the constraint of medical resources during the epidemic of COVID-19 [32], which may contribute to the occurrence of mental health problems for pregnant women in lockdown area. Moreover, lockdown measures disturb the normal daily routine, such as the reduction of physical excise and social life, which may influence the mental health status [33].

We also found that partial lockdown measures did not significantly increase the risk of prenatal depression. This also accords with earlier observations in the general population, which showed that the loosening of the restrictions is related to the decrease of mental health problems [6] and emotional eating [5]. The existing findings related to lockdown and mental health in pregnant women were inconsistent, which may be caused by different study designs and different interventions behind the relatively broad concept of “lockdown”. The implementation of lockdown measures in different countries varied, which means that the psychological consequences of these measures may be diverse. This result helps us to better understand how the current lockdown measures affect the mental health of pregnant women. It suggests that the use of differential closed-off management may minimize the “side-effect” of control measures on mental health among pregnant women, which is important for policy makers to adjust the lockdown measures and reduce the psychological consequences to the vulnerable people.

Compared to a study conducted during COVID-19 first outbreak using the same tool for mental health assessment in China [8], in this study, fewer pregnant women showed depressive symptoms during the COVID-19 rebound. A possible explanation for this might be that people learned how to deal with the COVID-19 related stress based on existing experience. Another possible explanation for this is that pregnant women got more social support compared to the first COVID-19 outbreak [34]. Government policies such as “community trio” (Additional file 1) were implemented to meet the basic need and provide psychosocial support for pregnant women. These results further support that more effective social support can reduce the risk of depression among pregnant women [10].

To our knowledge, this study is the first to report the quantitative relationship between geographic proximity to the COVID-19 patients and depression among pregnant women. A previous study in hazard proximity found that coastal border distance could significantly influence the perceived risk of earthquake/tsunami [35], while higher perceived risk was associated with increased level of anxiety [17]. In our study, a shorter distance to COVID-19 patients may represent a higher risk perception of infection, which may lead to the occurrence of mental health problems. The proximity to COVID-19 patients may also be related to a complex matrix of neighborhood attributes, such as the physical distance, land uses, residential density, neighborhood resources, and road transport. Future studies are needed to identify the important factors and their interactions behind this spatial scale.

There are several limitations in this study. First, the cross-sectional nature of the data could not determine the temporal link and support causal relationships between variables. Second, the published information on residence addresses of COVID-19 patients was not accurate to the house number because of privacy protection, so their geographic coordinates were estimated as the center position of the given location, which might lead to mis-estimation of the geographic proximity of pregnant women. Additionally, we excluded a small proportion of women without information on residence address, which might introduce potential biases on the studied association. Third, residual confounding may not be completely ruled out in the present study. Considering closed-off measures as the results of macro-decisions, we mainly adjusted for macro-factors including places of household registration and population density in the models. There are many factors which may be causally related to prenatal depression, including family structure and support, marital status, as well as prior mental health conditions [36, 37]. However, we believe these factors may not significantly distort the studied associations since they are not correlated with the exposure in the present study. Fourth, there might be possibility of response bias introduced by using self-rating questionnaire when pregnant women reported their personal experiences and symptoms. Moreover, the individual difference of knowledge about pandemic-related information, including the residence locations of COVID-19 cases, might lead to underestimation of the psychological effect of geographic proximity. However, excessive information seeking was a common behavior during the COVID-19 pandemic [38] , and one study had indicated that overwhelming majority of the people knew there were COVID-19 cases in the area where they live [39].

## Conclusions

Pregnant women who live close to COVID-19 patients have high risk of depression. A specific range around the residence of reported COVID-19 patients and the areas under strict closed-off management should be underlined as potential clustering of high depression levels in pregnant women. Our findings highlight the potential for early detection of pregnant women at a high risk of prenatal depression accompanying the prevention and control of ongoing COVID-19 epidemic or rebound, which may have policy implications for the mental health management during the epidemic of this and other similar infectious disease.

### Supplementary Information

**Additional file 1.** Detailed information of lockdown and partial lockdown measures.

**Additional file 2.** SDS scores and depressive symptoms under closed-off measures with different strictness at each trimester of gestation.

**Additional file 3.** Sensitivity analysis for association between geographic proximity and prenatal depression using logistic regression models.

## Data Availability

All data generated or analysed during this study are included in this published article and its supplementary information files.

## Abbreviations

COVID-19: Coronavirus disease 2019
SARS-CoV-2: Severe acute respiratory syndrome coronavirus 2
LMP: Last menstrual period
BMI: Body mass index
GIS: Geographic information system
SDS: Self-rating depression scale
OR: Odds ratio
CI: Confidence interval
