# Supplementary material for: Impact of closed-off management due to COVID-19 rebound on maternal depression during pregnancy
Source: BMC Pregnancy Childbirth. 2024 Jan 29;24:88. doi: 10.1186/s12884-024-06285-6 (PMC10823603; doi:10.1186/s12884-024-06285-6)
Supplement: Supplementary file 1 — Additional file 1. Detailed information of lockdown and partial lockdown measures. [file 12884_2024_6285_MOESM1_ESM.docx]

**Additional file 1 Detailed information of lockdown and partial lockdown measures**

|  | **Lockdown** | **Partial lockdown** |
| --- | --- | --- |
| **Community** | - Keep an entrance and exit guard 24 hours a day. People only can get in and can’t leave. - Vehicles are prohibited. | |
| **Commerce** | - Stop commerce and other group activity. | - Stop unnecessary commerce and other group activity. - Only 1-2 supermarkets are retained in the area and employees are not allowed to leave. |
| **Stay-home** | - Residents cannot leave their home. | - Residents cannot leave their home in principle. If they have completed nucleic acid tests twice with negative results, they can go to the community supermarket to buy something in an orderly way. |
| **Basic needs** | - Ensure the supply of water and electricity. - Encourage online shopping and contactless delivery. - Pay attention to the special needs of vulnerable group. | - Organize a community network to ensure the supply, and the supermarkets in community can provide services. - Storage points for take-out and express delivery can be appropriately set up, managed by a dedicated person. - Pay attention to the special needs of vulnerable people. |
| **Waste disposal** | - Waste generated by people in quarantine shall be clean everyday after disinfection. - Masks discarded after use by other residents shall be disposed in accordance with the requirements of household garbage. | - Waste generated by people in quarantine shall be cleaned every day, and designated garbage collection devices should be set up. |
| **Environmental disinfection** | - Disinfect the environment, especially in the key areas. | |
| **Health monitoring and services** | - Neighborhood committees and the police organize a health monitoring and service team. - The "community trio" (medical worker, police and civil servant) is responsible for the health monitoring of residents, and check their temperatures and symptoms twice a day. The people who have the symptoms such as fever and cough should be sent to designated hospitals. - Medical institutions in the area are responsible for the diagnosis and treatment of diseases of residents, and nucleic acid testing should be carried out in time when they need to be transferred outside the area. | |
| **Organized** **nucleic acid testing** | - All residents in the area will complete nucleic acid tests several times according to the order from authority (usually 5 times at 1^st^, 4^th^, 7^th^, 10^th^ and 14^th^ day of isolation). Avoid crowd gathering when sampling. | - All residents in the area will complete nucleic acid tests several times according to the order from authority (usually 4 times at 1^st^, 4^th^, 7^th^ and 14^th^ day of isolation). Avoid crowd gathering when sampling. |
| **Psychological guidance and maintenance of social order** | - Give the guidance to the public. - Maintain order and stability. - Strengthen psychological support. | |
| **Release of closed-off management** | - No new case reported for 14 days. - Effective control for cases and people who have close contact with cases. - Qualified environmental disinfection. - The community applies the release and the closed-off management will be released after district-level and city-level assessments. | |

Supplementary notice of Guangzhou coronavirus epidemic prevention and control headquarter on the classification of control. <http://www.gz.gov.cn/zt/qlyfdyyqfkyz/qktb/fygg/content/post_7309448.html>. Accessed 1 Jun 2021.
