# Supplementary material for: Impact of closed-off management due to COVID-19 rebound on maternal depression during pregnancy
Source: BMC Pregnancy Childbirth. 2024 Jan 29;24:88. doi: 10.1186/s12884-024-06285-6 (PMC10823603; doi:10.1186/s12884-024-06285-6)
Supplement: Supplementary file 2 — Additional file 2. SDS scores and depressive symptoms under closed-off measures with different strictness at each trimester of gestation. [file 12884_2024_6285_MOESM2_ESM.docx]

**Additional file 2 SDS scores and depressive symptoms under closed-off measures with different strictness at each trimester of gestation**

|  | **SDS scores (mean (SD))** | | | |  | **Depressive symptoms (*n* (%))** | | | |
| --- | --- | --- | --- | --- | --- | --- | --- | --- | --- |
|  | Total | 1^st^ trimester | 2^nd^ trimester | 3^rd^ trimester |  | Total | 1^st^ trimester | 2^nd^ trimester | 3^rd^ trimester |
| **Closed-off measures** | |  |  |  |  |  |  |  |  |
|  |  |  |  |  |  |  |  |  |  |
| Non-lockdown | 38.56 (8.54) | 39.33 (9.55) | 37.91 (8.25) | 39.00 (8.49) |  | 26 (4.96) | 6 (8.33) | 9 (3.88) | 11 (5.00) |
| Partial lockdown | 39.84 (9.12) | 40.81 (8.76) | 39.61 (9.55) | 39.73 (8.69) |  | 20 (8.93) | 3 (8.11) | 12 (10.53) | 5 (6.85) |
| Lockdown | 42.48 (10.48) | 42.52 (10.97) | 40.82 (9.19) | 44.86 (11.65) |  | 24 (18.18) | 6 (22.22) | 5 (8.06) | 13 (30.23) |
| *P* | <0.001 | 0.328 | 0.037 | 0.001 |  | <0.001 | 0.116 | 0.050 | <0.001 |

Abbreviation: SD, standard deviation
