# Supplementary material for: Impact of closed-off management due to COVID-19 rebound on maternal depression during pregnancy
Source: BMC Pregnancy Childbirth. 2024 Jan 29;24:88. doi: 10.1186/s12884-024-06285-6 (PMC10823603; doi:10.1186/s12884-024-06285-6)
Supplement: Supplementary file 3 — Additional file 3. Sensitivity analysis for association between geographic proximity and prenatal depression using logistic regression models. [file 12884_2024_6285_MOESM3_ESM.docx]

**Additional file 3 Sensitivity analysis for association between geographic proximity and prenatal depression using logistic regression models**

| **Geographic proximity (km)** | | | **n** | **Adjusted model ^a^** | |
| --- | --- | --- | --- | --- | --- |
|  |  |  |  | **OR** | **95% CI** |
|  | **Quintile 1** | **<0.77** | 177 | 10.59 | (2.25, 49.91) |
|  | **Quintile 2** | **(0.77-1.52)** | 175 | 9.41 | (1.97, 44.89) |
|  | **Quintile 3** | **(1.53-3.27)** | 176 | 9.36 | (1.97, 44.39) |
|  | **Quintile 4** | **(3.28-9.97)** | 177 | 2.62 | (0.61, 11.35) |
|  | **Quintile 5** | **≥9.98** | 175 | 1(ref.) | |

^a^ adjusted for age, pre-pregnancy BMI, gestational weeks, place of household registration and population density
